# Enigmatic Ladies of the Rings: How Cohesin Dysfunction Affects Myeloid Neoplasms Insurgence

**DOI:** 10.3389/fcell.2019.00021

**Published:** 2019-02-27

**Authors:** Alex Pezzotta, Mara Mazzola, Marco Spreafico, Anna Marozzi, Anna Pistocchi

**Affiliations:** Dipartimento di Biotecnologie Mediche e Medicina Traslazionale, Università degli Studi di Milano, Milan, Italy

**Keywords:** cohesin, AML, AMKL, MDS, animal models, gene expression

## Abstract

The genes of the cohesin complex exert different functions, ranging from the adhesion of sister chromatids during the cell cycle, DNA repair, gene expression and chromatin architecture remodeling. In recent years, the improvement of DNA sequencing technologies allows the identification of cohesin mutations in different tumors such as acute myeloid leukemia (AML), acute megakaryoblastic leukemia (AMKL), and myelodysplastic syndromes (MDS). However, the role of cohesin dysfunction in cancer insurgence remains elusive. In this regard, cells harboring cohesin mutations do not show any increase in aneuploidy that might explain their oncogenic activity, nor cohesin mutations are sufficient to induce myeloid neoplasms as they have to co-occur with other causative mutations such as *NPM1*, *FLT3-ITD*, and *DNMT3A*. Several works, also using animal models for cohesin haploinsufficiency, correlate cohesin activity with dysregulated expression of genes involved in myeloid development and differentiation. These evidences support the involvement of cohesin mutations in myeloid neoplasms.

## Cohesin Structure and Functions

**FIGURE 1:**
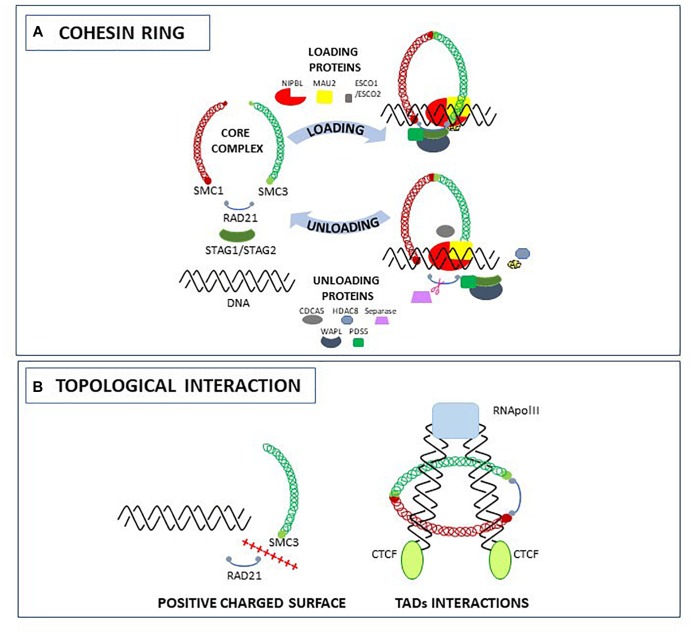
The cohesin complex and accessory proteins: structure **(A)** and topological interactions with the DNA **(B)**.

One of the biological meaning of a living organism is the possibility to divide by replicating DNA and generate a new organism. To accomplish this, the genome duplication should be error free and the daughter cell should properly inherit the genetic material from the mother cell. The cohesin proteins are required during this multistep process: in interphase to maintain genome stability during DNA double strand break repair (Watrin and Peters, 2009; Losada, 2014), in S-phase to enforce Sister Chromatid Cohesion (SCC) throughout DNA replication (Peters et al., 2008), and in M-phase to ensure proper chromosome distribution into dividing cells (Jeppsson et al., 2014). Since the cohesin protein complex has essential roles in the cell, members of the cohesin complex are found from bacteria to humans and are evolutionary and functionally conserved. The ability of cohesin to perform these functions resides in their property to encircle the DNA, creating topological links between chromatin fibers. To mediate sister chromatids tethering and segregation or DNA double strand break repair, the cohesin complex binds to the DNA in a *trans* conformation. However, cohesin might also encircle the DNA in *cis*, forming chromatin loops and contributing to gene regulation by modulating genome architecture or joining two distant segments of the genome. In vertebrates, the ring that embraces the DNA is formed by coiled-coil heterodimers of Structural Maintenance of Chromosomes (SMC) subunits SMC1 and SMC3, by the alpha-kleisin subunit RAD21 that brings in connection the ATPase head domains of SMC proteins and stabilizes their interactions (Nasmyth, 2011), and by the stromal antigens STAG1/STAG2 (SA1/SA2) (Canudas and Smith, 2009; Remeseiro et al., 2012). Although cohesin proteins are intrinsically able to topologically bind to the DNA (Gligoris et al., 2014), the loading of the complex is not efficient in the absence of the NIPBL/MAU2 heterodimer. As stated by their name, cohesin becomes “cohesive” only when the SMC3 head domain subunits are acetylated by the acetyl-transferase ESCO1/ESCO2. The release of the complex from the DNA is achieved by the separase-mediated proteolytic cleavage of RAD21 (Uhlmann et al., 2000), the HDAC8-mediated de-acetylation of SMC3, or the opening of the RAD21-SMC3 complex controlled by accessory proteins such as CDCA5 (soronin), PDS5 and WAPL (Ben-Shahar et al., 2008; Rowland et al., 2009; Nishiyama et al., 2010; Tedeschi et al., 2013; Beckouët et al., 2016; Figure 1A).

Recently, several studies were carried out to elucidate the role of the cohesin ring in topological entrapment of DNA. Biochemical and crystallographic studies in yeast led to the identification of an interlocking gate for the transportation of DNA across the cohesin ring (Murayama and Uhlmann, 2015), and of a specific HEAT-domain required for the binding of the DNA to the SMC3-RAD21 complex due to a positively charged surface (Li et al., 2018). Other studies using Chromosome Conformation Capture-derived approaches such as 5C and Hi-C, allowed the identification of a role for cohesin in the formation and stabilization of Topologically Associating Domains (TADs), genomic regions spanning form 200 kb to 1 Mb in mammals, that are thought to contribute to gene expression by remodeling chromosome architecture (Dixon et al., 2016). In a recent work using the smallest human chromosome 21, Bernardi demonstrated that the formation of TADs is related to the 3D structures of the corresponding GC-rich isochores. These “primary TADs” are bound to the cohesin complex that actively slides down, generating an extrusion loop (Bernardi, 2018). In another work it has been hypothesized that the movement of the cohesin complex along the extrusion loop is mediated by the pushing of the RNA polymerase (Björkegren and Baranello, 2018). Indeed, in *Drosophila* and mammals, cohesin can activate or silence genes by interacting with RNA polymerase II (RNA Pol II) (Misulovin et al., 2008; Schaaf et al., 2009; Fay et al., 2011). For example, by comparing *NIPBL* and RNA Pol II binding sites, it has been shown that NIPBL binds 100–200 nucleotides upstream of RNA Pol II (Zuin et al., 2014). The extrusion loop made by cohesin stops when DNA is occupied by the CCTC-binding factor (CTCF). In human and mammals, cohesin and CTCF co-localize at several loci contributing to topological organization of the genome: when CTCF is depleted, the cohesin complex is not found at CTCF sites (Wendt et al., 2008) but is still able to bind to other chromatin regions (Figure 1B).

Hence, cohesin proteins regulate both positively or negatively chromatin architecture and gene expression, by recognizing specific sites on the genome alone or in combination with different proteins, and modifying the interaction between enhancers and promoters.

## Cohesin and Tumourigenesis

Several evidences suggest that altered gene expression due to cohesin dysfunction could affect tumourigenesis. Indeed, cohesin or its regulators are frequently mutated in different types of tumors such as colorectal cancer (*NIPBL*) (Barber et al., 2008), glioblastoma, Ewing’s sarcoma, urothelial bladder carcinoma, melanoma (*STAG2*) (Solomon et al., 2011, 2013; Balbás-Martínez et al., 2013) and myeloid neoplasms (*STAG1*, *STAG2*, *SMC3*, and *RAD21*) (Dolnik et al., 2012; Kon et al., 2013). In addition, mutations in genomic binding sites of CTCF/cohesin (Katainen et al., 2015) or changes in cohesin protein levels (RAD21) have been associated to cancers (Yan et al., 2012). Since the cohesin complex is involved in chromosome segregation and DNA repair, it is not surprising that cohesin mutations or dysfunctions could enhance tumorigenesis. However, the association between cohesin mutations and genome instability in cancer is still controversial and not always reported (Balbás-Martínez et al., 2013; Taylor et al., 2014; Thol et al., 2014). To note, patients affected by the Cornelia de Lange Syndrome (CdLS) caused by germline mutations in cohesin genes (∼65% *NIPBL*, ∼5% *SMC1A* and *HDAC8*, 1–2% *SMC3* and *RAD21*) (Mannini et al., 2013; Singh and Gerton, 2015), rarely develop cancers and likely not for genomic instability but as a consequence of their clinical features (*e.g.*, gastric reflux) (Schrier et al., 2011; Deardorff et al., 2012). Since CdLS and tumors share same types of mutations in cohesin genes (for *NIPBL* heterozygous mutations, mainly non-sense, leading to haploinsufficiency; for *SMC1A* hemizygous mutations, mainly missense, probably leading to a dominant negative effect) (Mannini et al., 2013; Singh and Gerton, 2015), it has been hypothesized that they differ in their physiopathological contest. Indeed, in tumors somatic cohesin mutations occur in adult and terminally differentiated cells while in CdLS germline cohesin mutations occur in a developing and embryonic tissue. In addition, conversely to CdLS in which cohesin mutations are causative, in cancer cohesin mutations do not initiate but contribute to tumorigenesis when they co-occur with additional mutations. A study concerning AML identified 37 patients with mutation in one of the cohesin genes and, among them, the 81.1% had an additional mutation in genes causative for AML insurgence such as *FLT3-ITD* (21.6%), *NPM1* (21.6%)*, RUNX1* or *ASXL1* (Tsai et al., 2017).

## Cohesin Mutations in Myeloid Neoplasms

Acute myeloid leukemia (AML) is a heterogeneous group of hematologic aggressive neoplasms of bone marrow, characterized by irreversible expansion of precursor myeloid blasts defective in their differentiation and function (Löwenberg et al., 1999; Ley et al., 2008; Naoe and Kiyoi, 2013). The leukemic mutations are serially acquired in clones of long-lived self-renewing hematopoietic stem cells (HSCs), termed pre-leukemic HSCs (Jan and Majeti, 2013; Corces-Zimmerman and Majeti, 2014).

Among the novel recurrently mutated genes in AML patients, there are the members of the cohesin complex which occur in approximately 15% of AML cases (Thota et al., 2014). Interestingly, no correlation between mutated cohesin genes and prognosis was observed, and most of clinical features of AML patients with or without mutations in cohesin were similar (Thol et al., 2014). Thol and colleagues characterized the genomes of 389 uniformly treated AML patients in order to dissect the clinical and prognostic implications of mutated cohesin. A total of 23 patients (5.9%) had mutations in the cohesin genes and the most frequently mutated were *STAG1* (1.8%), *STAG2* (1.3%), and *SMC3* (1.3%), while mutations in *RAD21* and *SMC1A* were rarer events. Previously, The Cancer Genome Atlas identified mutations in cohesin in 26 out of 200 (13%) primary AML adult patients, with a higher mutations frequency in *STAG2*, *SMC1A*, *SMC3*, *RAD21* meanwhile no mutation in *STAG1* were observed in comparison to Thol and colleagues’ analysis (Ley et al., 2013; Thol et al., 2014). This discrepancy could be due to different approaches used to validate mutations (Thol et al., 2014). In myelodysplastic syndromes (MDS), a heterogeneous group of clonal hematopoietic disorders characterized by cytopenia, ineffective hematopoiesis and an increased risk of progression to AML (Haferlach et al., 2014; Shallis et al., 2018), *RAD21, STAG2*, and *SMC1A* are the most frequently mutated genes (∼15%) and are associated with poor survival (Cazzola et al., 2013; Kon et al., 2013; Haferlach et al., 2014; Malcovati et al., 2014; Thota et al., 2014). The identified cohesin mutations in AML patients are typically classified in two categories: mutations in *RAD21* and *STAG2* are mainly truncation or frame-shift while mutations in *SMC3* and *SMC1A* are mainly missense (Kon et al., 2013; Thota et al., 2014). Genomic deletions have also been found for *RAD21* and *STAG2* (Rocquain et al., 2010). Importantly, cohesin mutations are mutually exclusive suggesting that a single altered component is sufficient to affect the tumor suppressive function of the whole cohesin complex in myeloid leukemogenesis (Welch et al., 2012; Solomon et al., 2014).

The frequency of cohesin mutations is surprisingly higher in acute megakaryoblastic leukemia (AMKL), a rare sub-type of AML (AML-M7) characterized by defective megakaryocytes proliferation and differentiation (Ley et al., 2013; Yoshida et al., 2013). AMKL represents 4–15% of pediatric AML and is predominantly found in Down Syndrome children (DS) together with somatic *GATA1* mutations (Gruber and Downing, 2015). *GATA1* mutated isoforms cause the hyper-proliferation of megakaryocyte progenitors during early fetal liver hematopoiesis and lead to transient abnormal myelopoiesis (TAM) (Hitzler et al., 2003; Rainis et al., 2003). Up to 10% of TAM cases typically resolve spontaneously while 30% of them develop in AMKL during childhood with the accumulation of somatic mutations in different genomic regions (Rainis et al., 2003; Yoshida et al., 2013). Sequencing analysis revealed mutations and deletions in *STAG2*, *RAD21*, *SMC3, SMC1A*, and *NIPBL* in almost 53% of DS-AMKL patients but none in the TAM clones, indicating that cohesin mutations are involved in neoplastic transformation from TAM to AMKL. Only few non-DS-AMKL patients had cohesin mutations, suggesting that mutated cohesin is a DS-AMKL feature. Different works tried to dissect the effects of cohesin haploinsufficiency and altered GATA1 binding to the chromatin (Yoshida et al., 2013; Fisher et al., 2017). *GATA1* mutations were strictly linked to the context of trisomy 21 in DS patients (Shimizu et al., 2008) and this condition provided the cellular setting for the persistence and eventual transformation of *GATA1* mutant cells. Moreover, *GATA1* mutations involve a region that mediates GATA1-RUNX1 interaction during normal megakaryopoiesis (Elagib et al., 2003). Interestingly, the dosage effect caused by trisomy 21 hyper-activated the Wnt signaling in DS-AMKL patients. This is accomplished by the downregulation of the tumor suppressor gene *APC* and stabilization of the active form of beta-catenin via miR-99a/100-125 (Emmrich et al., 2014). The interaction between cohesin and Wnt/beta-catenin signaling has been described in different models of cancer (Ghiselli et al., 2003), and in our zebrafish model with cohesin haploinsufficiency (Pistocchi et al., 2013; Fazio et al., 2016; Mazzola et al., 2019), suggesting a possible effect of cohesin mutations and Wnt/beta-catenin signaling dysregulation in DS-AMKL.

## Cohesin, Gene Regulation and Animal Models

**FIGURE 2:**
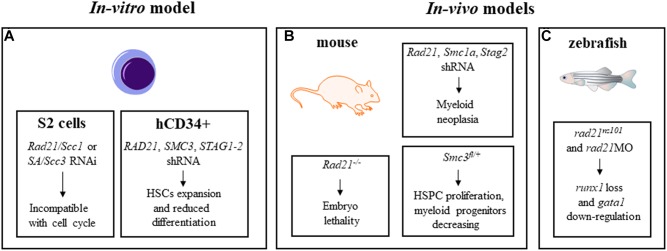
*In-vitro* **(A)** and *in-vivo* **(B,C)** models for the study of cohesin roles in hematopoiesis.

Cohesin mutations are associated to myeloid neoplasms, but are not causative of tumor onset indicating that other pathways might be relevant. Since cohesin mutations are often described as early, founder mutations in pre-leukemic HSCs (Corces-Zimmerman and Majeti, 2014), several groups investigated the role of cohesin complex in differentiation and self-renewal of HSCs and of their progenitor cells (HSPCs), and leukemogenic transformation using *in-vitro* and *in-vivo* models (Figures 2A–C).

Mullenders and colleagues described the role of cohesin in HSCs homeostasis (Mullenders et al., 2015). Firstly, they transduced murine bone marrow c-Kit^+^ HSPCs with shRNA against *Rad21*, *Smc3*, and *Smc1a* demonstrating a rapid increase of their replating capacity and defects in myeloid differentiation. Then, they generated a mouse model with cohesin down-regulation showing clinically features of myeloid neoplasia but not genomic alteration due to cohesin dysregulation. Moreover, they demonstrated that HSCs and myeloid precursors were subjected to changes in gene regulation and chromatin accessibility as a consequence of cohesin down-regulation (Mullenders et al., 2015). The correlation between the increase of hematopoietic progenitors and cohesin downregulation has been also described in a mouse model generated by Viny and colleagues. Cells derived from this mouse with combined effects of *Smc3* haploinsufficiency and *FLT3-ITD* mutation, showed increased HSPCs proliferation and survival rates and a simultaneous decrease in myeloid progenitors (Viny et al., 2015). The role of the cohesin complex as a major regulator of HSCs has also been described by Galeev and colleagues with genome wide RNAi analyses on primary human cord blood derived CD34^+^ cells with cohesin knock-down (Galeev et al., 2016). They demonstrated that in sorted CD34^+^ cells, the transfection of shRNA against *RAD21*, *STAG1-2* and *SMC3* negatively affected differentiation and enhanced HSCs expansion *in vitro* and *in vivo* when these cells were transplanted in immunodeficient mice (Figure 2B). Transcriptome analyses on cohesin-deficient CD34^+^ cells demonstrated an increased expression of genes responsible for the stem cell phenotype. Mazumdar and colleagues identified genomic regions with altered chromatin accessibility following *RAD21* or *SMC1A* mutations in primary human HSPCs. Using the transposase-accessible chromatin and sequencing (ATAC-seq) technique, they found increased accessibility of GATA2, RUNX1 and ERG DNA binding motifs when *RAD21* and *SMC1A* were mutated in comparison to controls. They also demonstrated that the block in the HSPCs differentiation was specifically due to the increased activity of these transcription factors as their shRNA-mediated silencing rescued the differentiation defects of cohesin-mutated-HSPCs (Mazumdar and Majeti, 2017) (Figure 2A). In a murine model, the loss of the *Asxl1* gene that is frequently mutated in different myeloid malignancies with poor prognosis, reduced the genome binding of *RAD21* and *SMC1A* and altered the expression of their target genes in cells enriched for myeloid progenitors Lin-cKit^+^ (LK) (Li et al., 2017).

*RUNX1*, an essential regulator of hematopoiesis frequently involved in leukemia insurgence, is also related to cohesin, as demonstrated by Horsfield and colleagues using a zebrafish model. The zebrafish *rad21^nz171^* mutant completely lacked early *runx1* expression in hematopoietic compartments, while monoallelic loss of *rad21* reduced the transcription of *runx1*, suggesting a dose-dependent modulation controlled by Rad21. Early hematopoiesis in *rad21^nz171^* mutant was not dramatically affected except for the *gata1* downregulated expression, while later differentiation was severely reduced, indicating that the altered expression of markers of late hematopoiesis depends on the loss of *runx1* and reduction of *gata1* (Horsfield et al., 2007; Figure 2C).

The hypothesis that cohesin dysfunction in HSPCs might alter the expression of hematopoietic genes was further investigated going inside the mechanism through which cohesin accomplish this. Fisher and colleagues demonstrated that cohesin complex binds to and regulates the Polycomb Repressive Complex 2 (PRC2) to silence *Hoxa7* and *Hoxa9* genes involved in HSPCs proliferation. Mutations in cohesin (*RAD21*) enhanced HSPCs proliferation but the phenotype could be rescued when *Hoxa7* or *Hoxa9* genes were simultaneously knocked-down (Fisher et al., 2017). The authors argued that the cohesin-mediated regulation of PRC2 interaction with *Hoxa* locus was accomplished through remodeling of chromatin architecture. Indeed, cohesin proteins interact with CTCF in DNA binding, specifically in the establishment of TADs (Merkenschlager and Nora, 2016; Bernardi, 2018). The binding of CTCF, together with the epigenetic chromatin-remodeling factor Smarca5 and cohesin was also found at upstream regulatory element (URE) of *SPI1* gene, a master transcription factor of myeloid cell differentiation. This recruitment was disrupted in AML blasts suggesting its involvement in tumor insurgence (Dluhosova et al., 2014). The specific action of the cohesin complex on myeloid cells was also observed in one case of gene fusion. Murine hematopoietic cells transfected with the fusion gene *NIPBL-HOXB9* exhibited increased *in vitro* colony replating capacity with hallmarks of myeloid progenitors (Dang et al., 2017). In our recent work, we used zebrafish to confirm that other mutations might dysregulate cohesin expression. In this regard, we observed that AML patients carrying *NPM1* mutations showed a specific *NIPBL* downregulation and a zebrafish model with *NIPBL* haploinsufficiency presented defects in myeloid cell differentiation, demonstrating that animal models could enhance the comprehension of the action of multiple mutations/dysregulations (Mazzola et al., 2019).

## Conclusion and Future Perspectives

In myeloid neoplasms cohesin mutations occur with low frequency in comparison to other more frequently mutated genes and are not sufficient, alone, to drive to tumourigenesis. However, cohesin mutations occur early in the clonal hierarchy and cohesin dysfunction enhances HSC and HSPCs proliferation and controls the expression of genes involved in myeloid differentiation. Therefore, cohesin might be considered as promising pharmacological target for myeloid malignancies. Some drugs already used in AML clinical trials such as Dot1I methyltransferase inhibitors (Bernt et al., 2011) or azacitidine, have been proved to be efficient in the rescue of the phenotype caused by cohesin mutations (Fisher et al., 2017; Tothova et al., 2017). Thus, the dissection of molecular pathways altered by cohesin dysfunction might allow the discovery of new therapeutic targets downstream of cohesin. In this regard, the screening for cohesin mutations of larger cohorts of patients or the development of animal models with cohesin haploinsufficiency are required to address this intriguing possibility. Moreover, the co-occurrence of mutations in cohesin and causative genes of myeloid neoplasms (*e.g.*, *FLT3-ITD*, *DNMT3A*, *NPM1, and TET2*), leads to the hypothesis that the efforts to develop therapies for AML might be improved by combining those targeting specific genes and those directed on shared targets, as well as by combining multiple therapies to treat diverse sub-clones.

## Author Contributions

AP, MM, and MS contributed to writing and figures. AM contributed to figures and supervision. AP conceived, wrote, and supervised the manuscript.

## Conflict of Interest Statement

The authors declare that the research was conducted in the absence of any commercial or financial relationships that could be construed as a potential conflict of interest.
